# Walrus history around the North Water: Human–animal relations in a long-term perspective

**DOI:** 10.1007/s13280-018-1027-x

**Published:** 2018-03-07

**Authors:** Anne Birgitte Gotfredsen, Martin Appelt, Kirsten Hastrup

**Affiliations:** 1 Natural History Museum of Denmark, University of Copenhagen, Øster Voldgade 5-7, 1350 Copenhagen, Denmark; 2 The National Museum of Denmark, Frederiksholms Kanal 12, 1220 Copenhagen, Denmark; 3 Department of Anthropology, University of Copenhagen, Øster Farimagsgade 5, 1353 Copenhagen, Denmark

**Keywords:** Archaeo-zoology, Atlantic walrus, Communal hunting, North Water polynya, *Odobenus rosmarus rosmarus*, Prehistoric sites

## Abstract

This article highlights the relationship between walruses and humans in and around the North Water polynya in a long-term perspective. The present study draws on a combination of biological, archaeological, archaeo-zoological, historical, and ethnographic sources covering the period from the 8th century ad to the late 20th century. The study demonstrates that the walrus was an important resource of meat, blubber, and other products throughout all the studied periods, if always supplemented by other kinds of game. It is suggested that walrus distribution and behaviour, as well as hunting strategies and technologies historically constituted a powerful component not only in forming human action and social life in the region but also in serving as an imaginative resource. It is further argued that the walrus and the walrus hunt still play a significant role in the present community living on the edge of the North Water, even if the hunt is increasingly circumscribed due to changing ice conditions.

## Introduction

In this paper, we explore the role of the Atlantic walrus (*Odobenus rosmarus rosmarus*) in the region around the North Water polynya (Pikialasorsuaq). The walrus has contributed significantly to the entire socio-ecological system in the region in shaping both the hunting economy and people’s aspirations, and offering up a rich source of meat and blubber—for people as well as dogs—of hides, ivory and imaginations through the ages. (e.g. Vibe 1950; Born 1987; Born et al. 1995, 2017 and references therein). The walrus also has its own history, so to speak—both in a long-term perspective (see Born 2005) and possibly from a shorter term perspective of change in overall distribution and movements (e.g. Stewart et al. 2014), and in its response to climate and hunting practices (e.g. Born 1987; Born et al. 2017). Both these time-scales impinge upon the human–animal relationships that are at the centre of this article.

More specifically, the article analyses the significance of the walrus as an agent in the economy, the social life, and the imageries of the hunting communities that have settled in the area through the ages. Even today, and in spite of increasingly difficult access due to changing movement patterns induced by climate change, walrus forms an important component of Inughuit hunting (Born et al. 2017), embodying the varied aspirations of people living both inside and outside Avanersuaq (the ‘big North’), often referred to as the Thule Region since early 20th century. As such the walrus has also become a site of contestation, some hunters insisting on their unlimited rights of access and on the sustainability of the traditional hunt, and marine biologists and management bodies wishing to ensure the long-term viability of walrus populations and walrus hunting on the basis of scientific measurements of the stock (e.g. NAMMCO 2006, 2010; Witting and Born 2014; Born et al. 2017).

**Fig. 1:**
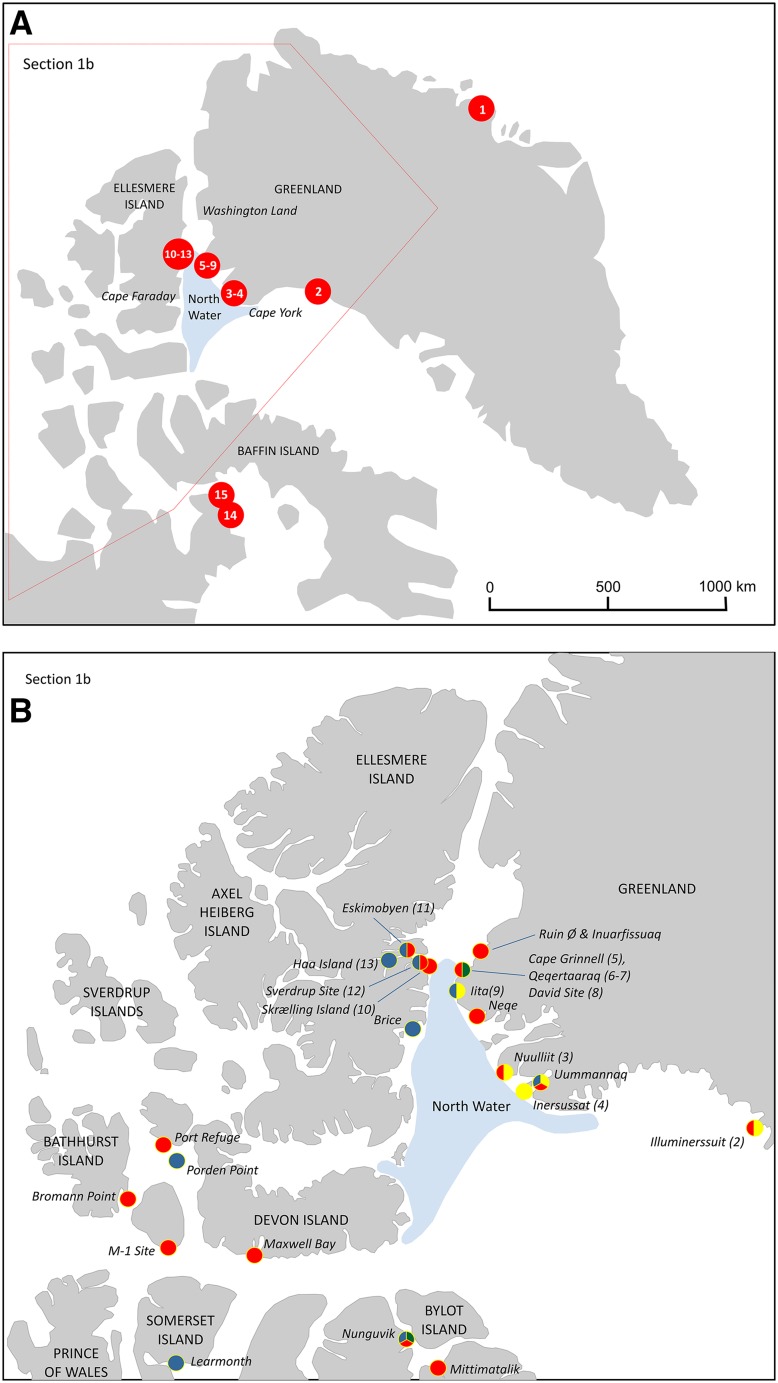
**a** Important archaeological sites mentioned in the text: (1) Hvalros Ø, (2) Illuminerssuit, (3) Nuulliit, (4) Inersussat, (5) Cape Grinnell, (6) SW-Point (Qeqertaaraq), (7) Walrus site (Qeqertaaraq), 8) David site, (9) Iita, (10) Skrælling Island, (11) Eskimobyen, (12) Sverdrup site, (13) Haa Island, (14) Sanirajak, (15) Alarnerk and the Igloolik Island. Numbers 1–14 refer to numbers in Table 1. Drawing: M. Appelt. **b** Important sites and their cultural components. Green dots = sites with late Dorset component, Red dots = sites with ruin Island-phase Inuit component, Blue dots = sites with post-Ruin Island-phase, Inuit component, and Yellow dots = Sites with Inughuit/Inuit component. Multi-colour dots indicate presence of more than one component on the site. Numbers in brackets correspond with numbers in (**a**). Drawing: M. Appelt

This article includes the results of new archaeological surveys and archaeo-zoological finds from the Thule Region as well as a reassessment of previous archaeological and historical sources. Based on this evidence and by including historical documentation we seek to understand how, when, and why the walrus has become emblematic in the perception of both the riches and the challenges of the living resources upon which social life in this High Arctic region always depended. The geographical emphasis is on the North Water region, which corresponds to the distribution of the northern Baffin Bay walrus stock as defined (NAMMCO 2010; Andersen et al. 2014; Stewart et al. 2014), in combination with the maximum extent of the lands that historically were used by people living in the North Water area, i.e. the sea ice- and landscape surrounding the North Water polynya from the tip of Cape York (Innaanganeq), north to Washington Land, across to the eastern coast of Ellesmere Island (Umingmak Nuna) and south to Cape Faraday (Fig. 1a, b). Comparisons will be made to relevant archaeological, historical and archaeo-zoological sources from the Foxe Basin, Central Canadian Arctic, and North East Greenland.

## Walrus life history, ecology, and distribution

Walruses are highly gregarious pinnipeds that tend to travel in small groups and haul out on ice or on land to rest, moult and bearing their young (Fay 1982). They inhabit the moving pack ice, or drift ice, and areas with thin-ice where they are capable of breaking through up to 20 cm thick ice and maintaining holes in even thicker ice. Although walruses may feed on a variety of bottom-dwelling invertebrates, only a few bivalves—usually *Mya* sp., *Hiatella* sp. and *Serripes* sp.—make up the bulk of their diet (Vibe 1950; Born 2003).

Recent assessments on walrus foraging behaviour in Smith Sound concluded that most dives for food were < 100 m [although much deeper dives were observed (Garde et al. 2018)], which coincided well with the bathymetric distribution of walrus preferred prey items i.e. the saltwater clams of the genera *Mya* and *Hiatella* (Vibe 1950). The patchy distribution of walrus is thus largely governed by ice coverage and thickness in combination with prey distribution (Born 2005). As most polynyas including the North Water have light see ice conditions during winter and shallow water banks with suitable walrus food, the walrus is a typical ‘polynya animal’ (Born 2005). This makes walruses an important and geographically predictable stable food and raw material source for the Inuit/Inughuit.

Sexual maturity is reached fairly late. For Atlantic walruses in the North Water the mean age of attainment of sexual maturity is c. 6 years of age in females and c. 11 years in males (Born 2001, 2003); the reproductive cycle is basically triennial with females giving birth every 3 years and the calf staying with its mother for at least 2 years (Born 2001). Generally, the capacity for population growth is considered to be relatively low (e.g. Born et al. 1995 and references therein), although annual maximum population growth rate up to 7–8% per year has been documented (Kovacs et al. 2014). In addition, walruses are vulnerable to environmental changes (e.g. Laidre et al. 2008), although it is disputed how and to what extent especially Atlantic walruses may be affected by climate change (cf., Born 2005; Born et al. 2017). Due to their restricted distribution walruses are relatively easy to hunt (Born et al. 1995, p. 9).

Presently, and presumably in former times, walruses together with belugas (*Delphinapterus leucas*), narwhals (*Monodon monoceros*), bearded seals (*Erignathus barbatus*) and ringed seals *(Phoca hispida*) winter in the general region of the North Water polynya, a large part of them in the eastern part of the polynya along the Greenland shores (Born et al. 2004; Heide-Jørgensen et al. 2016). During the open-water period in summer, the walrus distribution is more restricted and primarily confined to the eastern coast of Ellesmere Island, Canada. Walruses are therefore virtually absent during the open-water season in Greenland waters for a couple of months, except for a few stragglers (Stewart et al. 2014; Born et al. 2017, 56ff). In the North Water area, walruses are segregated on the basis of sex and age class, with females and calves to be found farther north than adult males (Vibe 1950; Born et al. 2017).

Walrus research from the last couple of decades has clearly demonstrated that in historic as well as modern times human societies have heavily influenced walrus populations, in terms of changed migration routes, population size reduction perhaps affecting population structure, and a shift in the distribution of the sex-segregated groups (e.g. Born et al. 1994, 1995; Wiig et al. 2007, p. 74; Witting and Born 2014). However, most recent studies indicate signs of recovery of the walrus subpopulation that are harvested in the North Water area (Witting and Born 2014). In the first half of the 20th century, there was allegedly an influx of walruses into the North Water from the south during June and July (Freuchen 1921; Vibe 1950). Recent aerial surveys could not confirm this migration pattern, however, which implies that migration routes may have shifted, as may the timing of migration (Born et al. 1994, 1995, 2017). Around the beginning of the 20th century, a terrestrial haul-out site, an *ulli*, at Littleton Island (Pikiuleq) and on the shore opposite the island still existed (Hayes 1867; Peary 1917; Freuchen 1921); in addition, Vibe was told in 1939–1940 about two haul outs in the Wolstenholme Fjord by a local hunter (Vibe 1950). The terrestrial haul outs on the Greenlandic side of the North Water are now all abandoned (Born et al. 1995, p. 52, 2017).

While the ringed seal constituted the cornerstone in subsistence of all prehistoric human populations in coastal Greenland, Arctic Canada, and Alaska (e.g. Murray 1996, 1999; Woollet et al. 2000), walrus was hunted and exploited by all coastal Inuit groups as well as their predecessors, although with varying intensity and in different ways at different periods. The ubiquitous ringed seal is accessible year round, whereas walruses and other large marine and terrestrial mammals have a more restricted distribution spatially and seasonally, and thus a varied accessibility. Ringed seal can be hunted, easily managed and transported by a single hunter. In contrast, the much larger walrus can be aggressive and will attack kayaks and *umiat* (open skin boats), or chase hunters through thin-ice from beneath; furthermore walrus females will protect their young with vigour (Nelson 1969, p. 363; Freeman 1975, p. 150; Fay 1982; Born et al. 2017). In other words hunting walrus for the hunter posed a high risk of becoming injured or even killed. Through a communal hunting strategy the risk of the individual hunter was minimised; along with the communal hunt a sharing system of the game developed within the community, distributing the meat more or less equally (e.g. Holtved 1967, pp. 116–122). Moreover, the fluctuating accessibility over the year warranted a well-developed storing system (e.g. Murray 1999, p. 476).

Basically, there are (and were) four ways of hunting walrus: First, hunting on thin-ice, while the ice is still so thin that the walrus can break through it to breathe and be harpooned by the hunter. This kind of hunt was practiced in early winter and in spring, although sometimes hampered or impeded when the snow cover became too thick (e.g. Vibe 1950; Born et al. 2017). Second, there is the hunting in pack ice, where walruses may haul out, and third, there was a hunting in open water from kayak (now motor-boat), performed during summer and early autumn. Finally, in regions where terrestrial haul-out sites were present the walruses were also targeted there (Born et al. 2017). While things have certainly not been ‘the same’ through the centuries, there are remarkable continuities, as we shall show in the following sections.

## Materials and methods

The archaeological and archaeo-zoological source material from the Greenlandic side of the North Water is substantial, reflecting 80 years of changing excavation practices. During the periods 1935–1937 and 1946–1947 Holtved (1944a, b, 1954) undertook the first systematic large-scale excavations in the area. In spite of excellent preservation conditions in the High Arctic, Holtved did not recover faunal remains (except for tool implements of organic matter). During the last couple of decades, Holtved’s work has been supplemented through excavation campaigns in 1996 and 1997 by the ‘Gateway to Greenland’ project at Hatherton Bay, Inglefield Land, which for the first time encompassed the collection of faunal material from the area, i.e. from four Late Dorset sites (Appelt and Gulløv 1999; Christensen 2000). Through archaeological surveys and excavations since the 1990s the ILAP (Ingefield Land Archaeological Project) project provided substantial new information and artefactual materials from both Late Dorset and Early Thule culture sites (Darwent et al. 2007, 2008; Darwent and Foin 2010; Darwent and Johansen 2010; LeMoine and Darwent 2010; Johansen 2013). Finally, the NOW Project 2014–2017 obtained new data on walrus exploitation from excavations at the classic Nuulliit site from midden layers in front of a *qassi* (a Men’s house) combined with comprehensive surface registrations of animal bones on sites in the Innaanganeq and the Pittufik area (Grønnow et al. 2015, 2016, 2017; Mønsted 2016). On the Canadian side of the North Water polynya extensive archaeological field-campaigns have been conducted since the 1970s notably in the central parts of eastern Ellesmere Island, covering all periods of the human history in the area. Both the archaeological and archaeo-zoological material from Ellesmere Island is very well-published and constitutes the central frame of reference for all of the North Water (McCullough 1989; Schledermann 1996; Schledermann and McCullough 2003).

Regarding the archaeo-zoological quantification methods the animal bones were quantified by NISP (number of identified specimens), which is normally considered the basic unit to measure taxonomic abundance among archaeo-zoological assemblages (e.g. Lyman 1996). Further the MNI (minimum number of individuals) were calculated in order to make the biomass [or TBM (total body mass)] estimations. The species identified and quantified walrus abundance, as expressed by NISP excluded the debitage, i.e. small splinters and fragments of tusk, penis bone (baculum), mandibles and maxillary bone resulting from tool production and tusk extraction, in order to make the number of walrus fragments comparable to the other game animals.

In addition to the prehistoric source material, this article also incorporate historical reports and archival material as well as more recent ethnographic sources. When it comes to the methods involved in analysing this follows well-established procedures for contextual interpretation and cross-cultural (or cross-temporal) comparison based on positive knowledge. In turn, such interpretive efforts widens the possible analysis also of prehistoric conditions, and allows for a more general conclusion of the walrus as a principal resource for humans, both past and present.

## The importance of walrus in prehistory

The prehistory of the North Water walrus hunting cannot be understood without including a wider geographical perspective. Therefore we include zoo-archaeological material from Foxe Basin, Central Canadian Arctic, where walruses form a stable year-round source (e.g. Born et al. 1995). The earliest specific cultural adaptations of walrus hunting is likely to have taken place among pre-Inuit Dorset groups in the Foxe Basin region (Murray 1996, 1999). The continued importance of the walrus hunting in the Foxe Basin is illustrated by including, for instance, the Ruin Island-phase site of Sanirajak (Desjardins 2013), which in several aspects resembled the abovementioned Nuulliit site. We will furthermore make reference to the post-Ruin Island archaeological record from Hvalros Ø (North East Greenland), as well as the newly analysed archaeological record from the Ruin Island-phase site of Illuminerssuit [Kap Seddon, Eastern Melville Bay (Qimusseriarsuaq)].

Dyke et al. (1999) examined walrus records across Arctic and Atlantic Canada and concluded that walrus remains occurred in scarce numbers in assemblages dating to early pre-Inuit cultures the so-called ‘Arctic small tool tradition’ or AST (Saqqaq, Independence, and Pre-Dorset) c. 2400–500 bc. They also concluded that walrus remains consisted primarily of debitage and not ‘diet-related’ remains (Dyke et al. 1999, 173f). On the Greenlandic shores of the North Water early pre-Inuit cultures are only sporadically evidenced and the meagre sites are devoid of preserved organic material (e.g. Sørensen 2010). The first substantial evidence of extensive walrus hunt in the eastern Arctic was seen among pre-Inuit Dorset groups (500 bc–500 ad) in the Igloolik area (Fig. 1a, no. 15), Canadian Foxe Basin (Murray 1996, 1999). An increase in the relative importance of walrus hunting (Murray 1999: Fig. 5; see also Dyke et al. 1999: Table 5) resulted in Dorset dwellings and their associated middens being considerably more substantial and numerous than the ruins of their predecessors (i.e. pre-Dorset cultures). This allowed for an increase of human population and a more sedentary life (Murray 1996, 1999). Interestingly, communal hunting activity was evidenced by an increase in walrus hunting harpoon heads being decorated, probably marking ownership of game and facilitating the relationships between hunters and their sharing of the walrus (Murray 1999, 474f). The Pre-Inuit Late Dorset sites located at the edge of the North Water (c. 700–1300 ad) exhibited a similar pattern, i.e. with heavier dwelling structures and deeper middens, likely the result of less mobility in the area, with (communal) walrus hunting playing an important role in the economy, social organisation and worldview (Schledermann 1990; Appelt and Gulløv 1999; Christensen 2000).

**Fig. 2:**
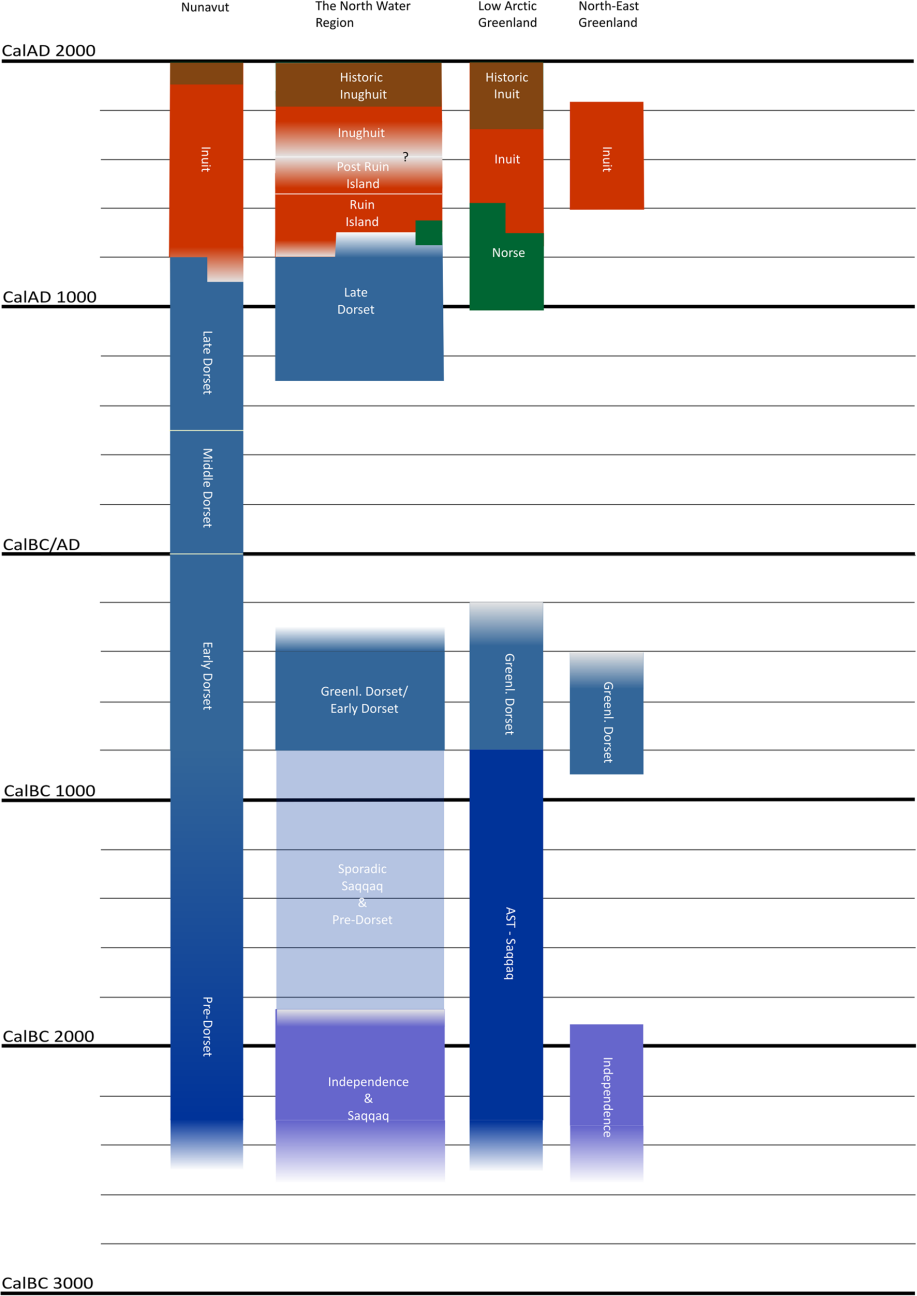
The chronological framework. Modified after M. Appelt in Maanasa et al. (2014)

Generally, the long-term history of human presence in the NOW-area is punctuated, with periods of possibly 200–500 years of continuous presence succeeded by periods ranging from 100 to 1000 years with limited occasional or no human presence in the area (Figs. 1a, b, 2). In the following paragraphs all dates are given in calibrated dates unless otherwise stated. In the present context, the chronological starting point is defined by the pre-Inuit Late Dorset (sometimes known as Tunit) groups’ settling around the North Water sometime during the 8th century ad. The presence of Late Dorset groups in the area seems to have been more or less continuous until their disappearance during the late 13th century ad (e.g. Appelt and Gulløv 2009). This happened synchronously with and partly connected to, the earliest Bering Strait Inuit moving into the eastern Arctic sometime in the 13th century ad (Friesen and Mason 2016; see also Hastrup et al. 2018). The early phase is known as the Ruin Island-phase; its geographical extent is not entirely clear, but by the 14th century ad Inuit sites are well-established from the northern Melville Bay north to Hall Land and along the central part of eastern Ellesmere Island (Holtved 1944a, b, 1954; McCullough 1989). The architectural layout and the archaeological assemblages uncovered from the 10 known Ruin Island-phase Inuit sites in the NOW-area (Fig. 1b) clearly indicate that communities were organised around communal hunting of baleen whales and walrus, headed by the *umiaq* owners—the *umialik*—architecturally manifested in the so-called Men’s house and likely involving crews from all households in the villages (e.g. Savelle 2002; Mønsted 2016).

From the early 15th century a considerable part of the human population probably moved out of the NOW-area and settled in North East Greenland (Sørensen and Gulløv 2012). At the same time, or perhaps beginning a century earlier, other Inuit groups moved south and settled in all the coastal areas south of the Melville Bay, while some moved northeast and down south again along the East Greenlandic coast (Gulløv 2004). In the following, the Inuit settlements in the NOW-area from the middle of the 15th century until the early 17th century will be referred to as the post-Ruin Island-phase. After mid-15th century ad there is no evidence of Men’s houses still in use in the NOW-area and only limited evidence for hunting of large whales and for the use of the *umiaq*. It is likely that the *umialik*-institution was dissolving in the beginning of the post-Ruin Island-phase.

The walrus hunt seems to have continued to play a crucial role during the post-Ruin Island-phase; even if there is no direct evidence of a communal hunt, it was most likely the case given the strength needed for manoeuvring the huge animal. While the richness of the North Water ecosystem was capable of supporting an Alaskan lifestyle of early Inuit groups for a couple of centuries, based equally on whale and walrus hunting, it collapsed during the 14th–15th century. Archaeological sites dated to the 16th and 17th centuries are few in the NOW-area and probably the human population in the area was at a very low level during these centuries (Schledermann and McCullough 2003). The few known sites, such as Iita (or Etah), are found at locations with direct access to stable and predictable hunting, e.g. of little auk (*Alle alle*) colonies, Arctic fox (*Alopex lagopus*), and walrus (LeMoine and Darwent 2016). The expansion of the Inuit population in the larger Thule area is likely to have taken place in the 18th century ad (Schledermann and McCullough 2003).

**Fig. 3:**
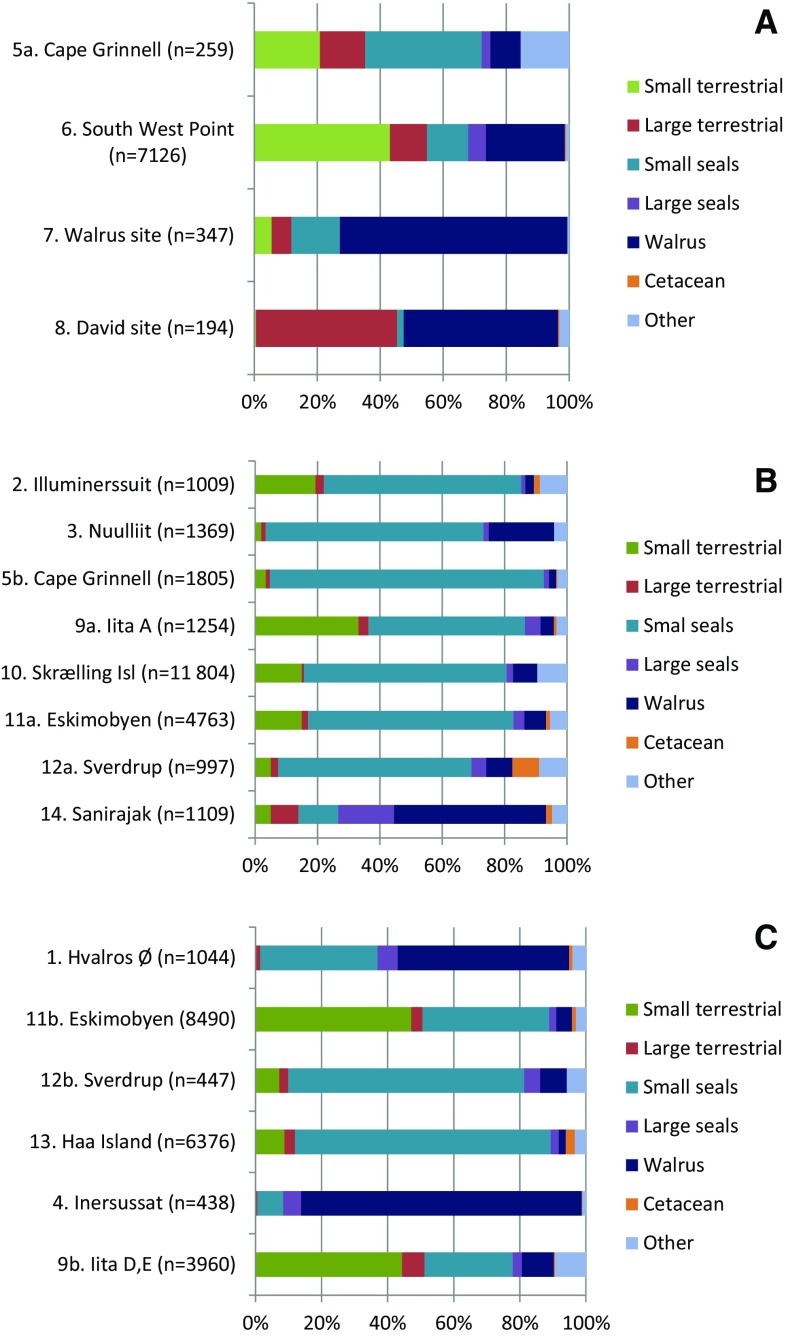
Census of the relative frequencies of walrus and other mammalian game at the studied sites based on NISP (number of identified specimens) counts. **a** Late Dorset, **b** ruin Island-phase, **c** post-Ruin Island-phase (nos.: 1, 11b, 12b and 13) and Inughuit period (nos.: 4 and 9b). Data from Table 2

In order to trace back in time the significance of walrus hunting in the North Water area based on the archaeological evidence a number of faunal bone assemblages were studied (Fig. 1a, b and Table 1). Walrus abundance as measured by NISP counts varied greatly across the studied sites and across the chronological periods (Table 2 and Fig. 3a–c). It is, however, important to note that not all sites were directly comparable due to a variable number of archaeological structures, e.g. middens, turf-houses and meat caches, at each site and therefore of variable sample sizes (see Tables 1, 2).

**Table 1:** Prehistoric sites included in the present study. Site numbers 1–14 refer to numbers on the map Fig. 1a and b. NISP (number of identified specimens) is given for each site of the total number of bones of fish (F), birds (B) and mammals (M), respectively. ^a^Only species identified mammal bones included due to a large amount of tusk and bone debitage. ^b^The bones were recorded by surface in situ registration and not excavated

Site no.	Site	Structures	Culture	Dating	NISP (F:B:M)	Reference
1	Hvalros Ø^b^	Tent rings, meat caches, shelters	Post-Ruin Island-phase	15th–mid-19th century	(0:29:1145)	Grønnow et al. (2011)
2	Illuminerssuit	F3 & 4, double turf house & midden	Ruin Island-phase	14th century	(0:14:2293)	Gotfredsen, unpubl.
3	Nuulliit^a^	F-Holtved 30 *qassi*, midden	Ruin Island-phase	14th century	(0:335:1369)	Grønnow et al. (2016)
4	Inersussat^b^	Tent rings, meat caches	Inughuit	c. AD 1900	(0:50:464)	Grønnow et al. (2017)
5a	Cape Grinnell	F88, turf house	Late Dorset	13th–14th century	(12:65:346)	Darwent and Foin (2010)
5b	Cape Grinnell	F20, turf house	Ruin Island-phase	13th–early 15th century	(2:82:2360)	Darwent and Foin (2010)
6	South West Point	F1, 2 & 4, turf houses & middens	Late Dorset	11th–mid-13th century	(0:2620:7220)	Christensen (2000)
7	Walrus Site	F-161, turf house & midden	Late Dorset	mid-12th–14th Century	(0:14:350)	Christensen (2000)
8	David Site	Arctic Megalith, midden	Late Dorset	7th–12th century	(0:539:194)	Christensen (2000)
9a	Iita	Turf house	Ruin Island-phase (period A)	15th century	(1:577:1506)	Johansen (2013)
9b	Iita	F1 & 2, turf houses & middens	Inughuit (period C & D)	mid-19th–early 20th century	(5:14 553:5087)	Johansen (2013)
10	Skrælling Island	F1–23, turf houses	Ruin Island-phase	12th–14th century	(0:149:11 804)	McCullough (1989)
11a	Eskimobyen	F1, 25 & 26	Ruin Island-phase	12th–13th century	(0:301:4763)	McCullough (1989)
11b	Eskimobyen	F23, 7 & 8, turf houses	post-Ruin Island-phase	15th–17th century	(0:336:8490)	McCullough (1989)
12a	Sverdrup	F6-8, turf houses	Ruin Island-phase	14th century	(0:11:997)	McCullough (1989)
12b	Sverdrup	F2 & 20, turf houses	post-Ruin Island-phase	15th–17th century	(1:12:447)	McCullough (1989)
13	Haa Island	F9-11, 17, 19 & 24, turf houses	post-Ruin Island-phase	15th–17th century	(0:36:6376)	McCullough (1989)
14	Sanirajak	F10 & 12-16, middens	Ruin Island-phase	13th–15th century	(0:159:2794)	Desjardins (2013)

**Table 2:** Census of the relative frequencies of mammalian game. Small terrestrial: Arctic hare and Arctic fox; large terrestrial: caribou and muskox; small seals: mainly ringed seal; large seal: mainly bearded seal: Cetacean: large whales; other: mainly dog/wolf, polar bear and monodontid whales. In case a taxonomic group was designated Phocidae, NISP was distributed on small seals and large seals proportionately; small terrestrial were lumped with small terrestrial; Artiodactyls with large terrestrial and pinnipeds were lumped with other. All NISP counts of walrus excluded debitage of tusk, maxillary bone and baculum. ^a^MNI for surface recorded included. Number of structures (N Feature) producing the bone assemblage and variation of walrus MNI (min–max) within structures are shown

Late Dorset	5a. Cape Grinnell		6. South West Point		7. Walrus site		8. David site	
TAXON	NISP	%NISP	NISP	%NISP	NISP	%NISP	NISP	%NISP
Small terrestrial	54	20.9	3066	43	19	5.5	1	0.5
Large terrestrial	37	14.3	842	11.8	22	6.3	87	44.8
Small seals	96	37.1	931	13.1	53	15.3	4	2.1
Large seals	7	2.7	399	5.6		0		0
Walrus	25	9.7	1789	25.1	251	72.3	95	49
Cetacean		0	21	0.3		0	1	0.5
Other	40	15.4	78	1.1	2	0.6	6	3.1
Total NISP, mammals	259		7126		347		194	
N Feature	1		5		2		4	
MNI walrus (min–max)	2		1–16		30^a^		18^a^	

Ruin Island-phase	2. Illuminerssuit		3. Nuulliit		5b. Cape Grinnell		9a. Iita, A		10. Skrælling Isl.		11a. Eskimobyen		12a. Sverdrup		14. Sanirajak	
TAXON	NISP	%NISP	NISP	%NISP	NISP	%NISP	NISP	%NISP	NISP	%NISP	NISP	%NISP	NISP	%NISP	NISP	%NISP
Small terrestrial	195	19.3	27	2.0	60	3.3	415	33.1	1762	14.9	710	14.9	50	5.0	55	5.0
Large terrestrial	27	2.7	20	1.5	25	1.4	41	3.3	83	0.7	100	2.1	23	2.3	99	8.9
Small seals	638	63.2	956	69.8	1586	87.9	629	50.2	7672	65.0	3132	65.8	618	62.0	141	12.7
Large seals	11	1.1	23	1.7	31	1.7	63	5.0	245	2.1	165	3.5	47	4.7	199	17.9
Walrus	38	3.8	287	21.0	40	2.2	53	4.2	923	7.8	335	7.0	84	8.4	540	48.7
Cetacean	13	1.3		0.00	7	0.4	10	0.8	0	0.0	59	1.2	86	8.6	22	2.0
Other	87	8.6	56	4.1	56	3.1	43	3.4	1119	9.5	262	5.5	89	8.9	53	4.8
Total NISP, mammals	1009		1369		1805		1254		11 804		4763		997		1109	
N Feature	2		1		1				23		3		3		6	
MNI walrus (min–max)	1–3		8		3				1–7		2–4		2		2–8	

Post-Ruin Island-phase	1. Hvalros Ø		11b. Eskimobyen		12b. Sverdrup		13. Haa Island		4. Inersussat		9b. Iita D,E	
TAXON	NISP	%NISP	NISP	%NISP	NISP	%NISP	NISP	%NISP	NISP	%NISP	NISP	%NISP
Small terrestrial	1	0.1	4001	47.1	32	7.16	551	8.6	0	0.0	1754	44.3
Large terrestrial	14	1.3	286	3.4	12	2.68	211	3.3	2	0.5	269	6.8
Small seals	371	35.5	3251	38.3	319	71.36	4930	77.3	34	7.8	1058	26.7
Large seals	64	6.1	186	2.2	22	4.92	158	2.5	24	5.5	112	2.8
Walrus	541	51.8	403	4.8	36	8.05	134	2.1	372	84.9	383	9.7
Cetacean	10	1.0	99	1.2	0	0	172	2.7	1	0.2	14	0.4
Other	43	4.1	264	3.1	26	5.82	220	3.5	5	1.1	370	9.3
Total NISP, mammals	1044		8490		447		6376		438		3960	
N Feature	1000+		3		2		6		262			
MNI walrus (min–max)	21		2–5		1–2		1–3		22^a^

At the pre-Inuit Late Dorset sites walrus comprised between 10 and 72% of the mammals with the Hatherton Bay sites (nos. 6–8) varying between 25 and 72%. This is quite a substantial amount of walrus compared to other High Arctic Late Dorset samples reported to amount to less than 5% (e.g. Darwent and LeMoine 1998; Schledermann 1990). At the Ruin Island-phase Inuit sites walrus comprised between 2 and 49% with the Sanirajak site (no. 14) in Foxe Basin, Central Canadian Arctic amounting to c. 49%, whereas the remainder varied between 2 and 21%. Finally at the post-Ruin Island-phase Inuit sites including the Inughuit period walrus varied between 2 and 85%. However, by leaving out Inersussat on Saunders Island (Appat) and Hvalros Ø, North East Greenland, the post Ruin Island-phase sites varied between 2 and 10%. Saunders Island was contemporary with the sites of Neqe and Pitoqavik and known as focal places for walrus hunting, also in historical times (Vibe 1950; Grønnow 2016 and references herein; Born et al. 2017). Besides being pivotal places for walrus hunting Inersussat at Saunders Island and Hvalros Ø situated by another recurring polynya the Sirius Water, East Greenland (Gotfredsen 2010; Grønnow et al. 2011) were recorded in the same way by surface in situ registration and hence differently from the remainder of sites. This surface registration method may have affected the recorded high relative importance of walruses.

**Fig. 4:**
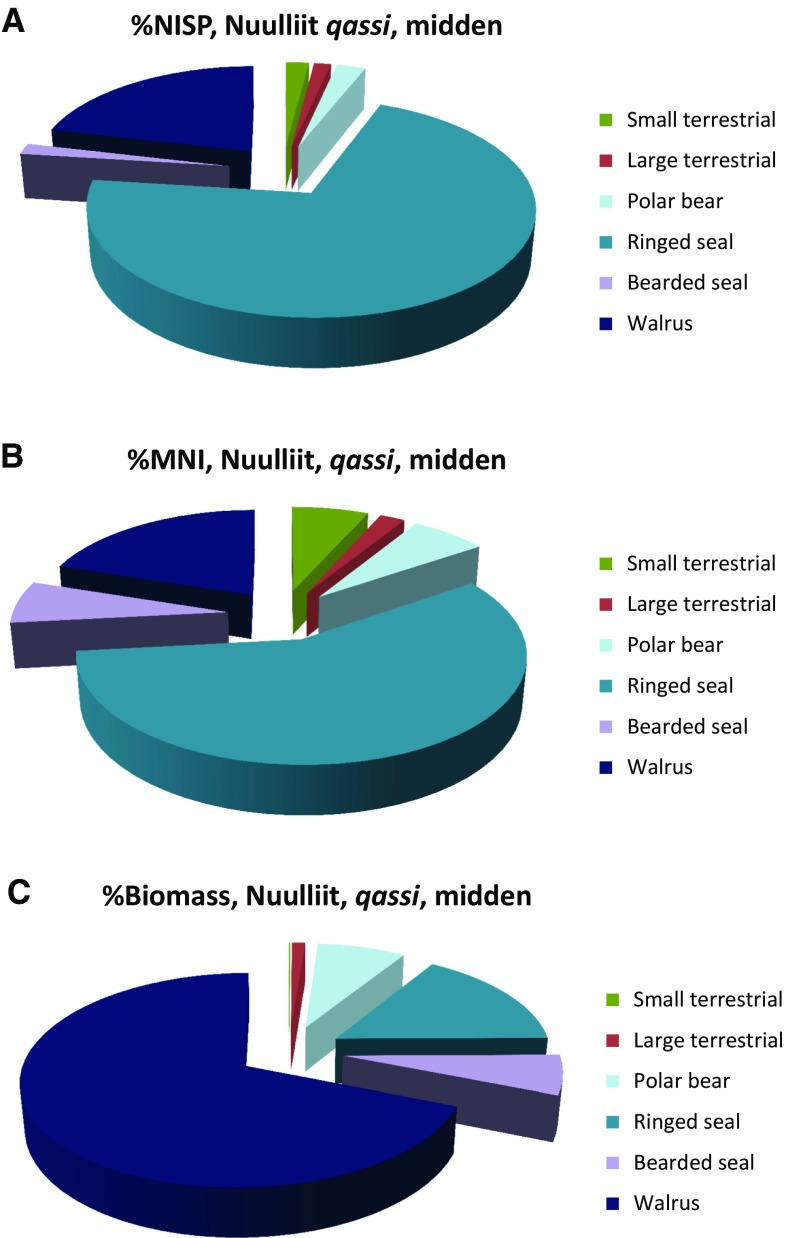
Census of the relative frequencies of the species identified portion of mammal bones at the Nuulliit, *qassi* midden. The relative importance of the various species changes dramatically whether quantified in terms of **a** NISP (number of identified specimens), **b** MNI (minimum number of individuals) and **c** biomass. Data from Tables 2 and 4

Through all the studied cultural periods the walrus ranked between 1 and 4 in the archaeo-zoological assemblages, as expressed by NISP based abundance, except for the post Ruin Island-phase Haa Island site (no. 13) where it ranked 6 (Table 3). Small seals, almost exclusively consisting of ringed seal, formed the staple resource and ranked 1 in terms of NISP as well as MNI at the majority of sites. For instance, at the Eskimobyen site MNI of small seals varied between three and 32 among the excavated structures (*n* = 9), whereas walrus varied between one and five (Schlederman and McCullough 2003). However, when one accounts for the amount of meat, blubber and other products such as hide and tusk provided per walrus the importance shifted in favour of walrus and other large sized marine mammals (see also Gotfredsen 2010). Adult females of Atlantic walruses reach an average total body mass (TBM) of 700–800 kg (Knutsen and Born 1994), whereas male reach a TBM of 1100–1200 kg, some may even reach 1600 kg (Knutsen and Born 1994; Born and Aquarone 2007) opposed to the adult ringed seal reaching c. 55 kg (Born and Böcher 2001). At the *qassi* midden on the Nuulliit site (no. 3) the distribution of the species identified mammals clearly illustrates to which extent the large sized marine mammals contributed to the (food) economy when quantified in terms of biomass as expressed by the total body mass (TBM) opposed to NISP (Fig. 4a–c). How much the various species actually contributed to the food economy is difficult to estimate. There may have been a considerably seasonal and inter-annual variability. Born (1987) estimated that walrus annually provided c. 25% of edibles mainly from open-water hunting in the Thule area. Estimations were based on information gathered from hunters of the Thule area between 1977 and 1985 (Born 1987). The estimated amount of biomass contributed by bowhead whales (*Balaena mysticetus*) was left out of the calculations since it would have completely overshadowed the importance of other species. Still, the remains of at least four individual bowhead whales were present at the Nuulliit peninsula all of which were presumed to belong to the Ruin Island-phase. One of the skulls located adjacent to the excavated *qassi* midden was radio-carbon dated to 1302 ± BP (AAR-24771) and thus dated to ad 1056-1143 (1 Sigma range; δ13C = − 14.49 ± 0.23) (Stuiver and Reimer 1993). Due to variability in the marine correction reservoir age we consider the bowhead skull to be contemporary with the habitation of the site.^1^ Given that all bowheads were contemporary they would have contributed massively to the resource influx of the Ruin Island population.

**Table 3:** Rank order of the four most common taxa of the studied sites. ^a^Taxonomic richness (Ntaxa) was given for mammals only

TAXA	Late Dorset					Ruin Island-phase							Post-Ruin Island-phase				Inughuit	
	5a	6^a^	7^a^	8^a^	10	2	3	5b	9a	11a	12a	15	1	11b	12b	13	4	9b
Small terrestrial	2	1			2	2		2	2	2				1	3	2		1
Large terrestrial	3	3	2	3												3		
Polar bear							3											
Small seals	1		3	2	1	1	1	1	1	1	1	3	2	2	1	1	2	2
Large seals									3			2	3				3	
Walrus	4	2	1	1	3	4	2	3	4	3	2	1	1	3	2	6	1	3
Cetacean											3							
Monodontids						3												
(Ntaxa)	14	12	7	7	23	10	16	13	16	21	15	12	14	14	11	12	12	17
NISP total	423	8297	730	286	15 237	2307	1704	2444	2084	5219	1234	2953	1174	15 871	577	9191	514	19 645

**Table 4:** Census of mammals from Nuulliit, the *qassi* midden. Total body mass (TBM) average mean weights are for arctic hare from Vibe (1981), for arctic fox from Müller (1906), for polar bear and walrus from Born (1987) and for bearded seal, adult ringed seal and bowhead whale from Born and Böcher (2001, Fig. 5.124), while weights for young ringed seals are from (Smith 1973). MNI (minimum number of individuals). Data of the Nuulliit midden from Gotfredsen (unpublished)

Taxon	Sex/age group	Total body mass (TBM) in kg	MNI	Excl. bowhead whale		
				%MNI	Biomass	%Biomass
Arctic fox		3	2	4.44	6	0.08
Arctic hare		3.55	1	2.22	3.55	0.05
Caribou	Average	87.5	1	2.22	87.5	1.12
Polar bear	Average	200	3	6.67	600	7.67
Ringed seal	Subadult	35	9	20.00	315	4.02
Ringed seal	Adult, average	55	17	37.78	935	11.95
Bearded seal	Average	160	3	6.67	480	6.13
Walrus	Average	600	9	20.00	5400	68.99
Total			45	100	7827.05	
Bowhead whale	Average	40 000	1			

Information on the sex and age distribution of the hunted walruses is crucial knowledge since the demographic profile of the catch influences productivity of the walrus population (see Witting and Born 2014). Even if high resolution sex and age profiles based on the archaeo-zoological assemblages are difficult to obtain there are some obvious trends. The Ruin Island-phase Nuulliit *qassi* sample of walrus tusks, representing at least eight individuals, provided three (c. 20%) specimens with traces of enamel still covering their tusk tip. According to King (1983) enamel caps are worn off at an age of c. 2 years, implying that in the Nulliit sample there were walrus calves caught around the time of weaning. Moreover, the historic Inughuit site Inersussat at Saundes Island provided epiphyseal fusion data indicating that slightly more than a third were hunted as juvenile or subadult (of nearly adult size) and still sexually immature walruses (Gotfredsen unpublished data).

Walruses are characterized by a considerable sexual dimorphism, allowing sex profiles to be established by measuring relevant skeletal elements such as mandibles (e.g. Wiig et al. 2007). The measured mandibles (*n* = 19) from Inersussat showed two clusters, most likely representing females and males respectively, with females outnumbering males (Gotfredsen, unpublished data). Only two mandibles could be measured from the Nuulliit *qassi* midden both likely deriving from females. While limited, the biological remains suggest that all age groups and both sexes were hunted by prehistoric people (see also Monchot et al. 2013). This hunting pattern differs from the present-day situation. Especially after the introduction of quotas in 2006 the walrus catches tend to be skewed towards large adult males, providing larger amounts of meat (to feed the dog teams) and bigger trophies, although some hunters still prefer younger walruses and/or adult females due to their more tender meat (Born et al. 2017, pp. 47, 81).

Still, the mere amount of debitage at some sites, as for instance Nuulliit, where the ivory debitage comprised c. 20% and walrus bone debitage c. 7% of the total number of faunal remains, points to the importance of walrus as a source of raw material and not only edible products. Further, as seen across the material collected by Holtved during his 1947-excavation (Holtved 1954) of most of the dwellings (50+ dwelling structures) at Nuulliit 34% of all tool types involve walrus tusk, or when viewed on individual specimens 140 of 565 (25%) are made of walrus tusk. At the Ruin Island-phase Inuit site Sanirajak, Desjardins (2013, p. 46) similarly reported on ivory debitage to occur abundantly through all midden layers and recovery of a large number of ivory artefacts. Already in the pre-Inuit Dorset and Late Dorset cultures walrus tusk and bones formed an important resource for tool making often outnumbering other sources (e.g. Darwent and LeMoine 1998; Monchot et al. 2013). These finds attest to the significance of the walrus across cultures as a source of food for humans and dogs alike, of fuel, and of materials for the production of tools and hunting weapons.

Walrus hunting always depends on information, on available technologies (hunting tools and means of transportation), and timing, which again are connected to an entangled web of cultural historical, social, ideological and economic factors. When arriving in Avanersuaq in the 8th century ad the pre-Inuit Late Dorset seem to have depended on communal walrus hunting (Murray 1996; Appelt 2004) from the ice (whether on thin-ice or in ice-leads) and hunting at terrestrial haul-outs. We have no archaeological evidence of any sophicated means of transportation such as the types of vessels used later by Inuit (Appelt et al. 2016, pp. 786–787) that would have permitted open-water hunting. During the Ruin Island-phase, Inuit hunting technology included the use of both *kayak* and *umiaq*, adding open-water hunting to the range of possibilities in the region, and the use of the newly introduced dog-sledge would have made transport of people, information, and food across the region faster and more reliable.

When Europeans encountered the Inughuit living in the region in early 19th century ad, the use of both *kayaks* and *umiaqs* had fallen out use—not only restricting the warm-season mobility of the Inughuit communities, but also removing the possibility of open-water hunting (see e.g. LeMoine and Darwent 2016). With the arrival of a group of Baffin Islanders to Avanersuaq in the early 1860s (e.g. Mary-Rousseliere 2002) the *kayak* was one of the technologies that was re-introduced to the Inughuit, effectively leading to improved food security as reflected in what seems to be a rising population of Inughuit (e.g. Gilberg 1976).

## The imaginative significance of walrus across centuries

In many indigenous societies across the Arctic personhood is attributed to animals (e.g. Nuttall 2000; Hill 2011). In other words animals, especially important game animals such as caribou and marine mammals, can be perceived as other-than-human-persons (Hill 2011, p. 407). The human–animal dynamics in societies that understand animals as persons are based on mutual respect and the concept of reciprocity (e.g. Fienup-Riordan 1994, pp. 58–59; Jordan 2008, pp. 236–239). Important game animals and humans were ‘kinsfolk’ so to say and could in many aspects behave in similar ways (Hill 2011 and references herein). The archaeological remains resulting from the human concept of human–walrus relations could be expressed in various ways. Some were listed by Hill (2011, 412ff) from Alaska and Chukotka; comprising different kinds of amulets, large skull features with numerous walrus skulls in mounds or circles but also bone caches and ‘shrines’. In the following some archaeological and ethnographic examples across cultures will be presented.

**Fig. 5:**
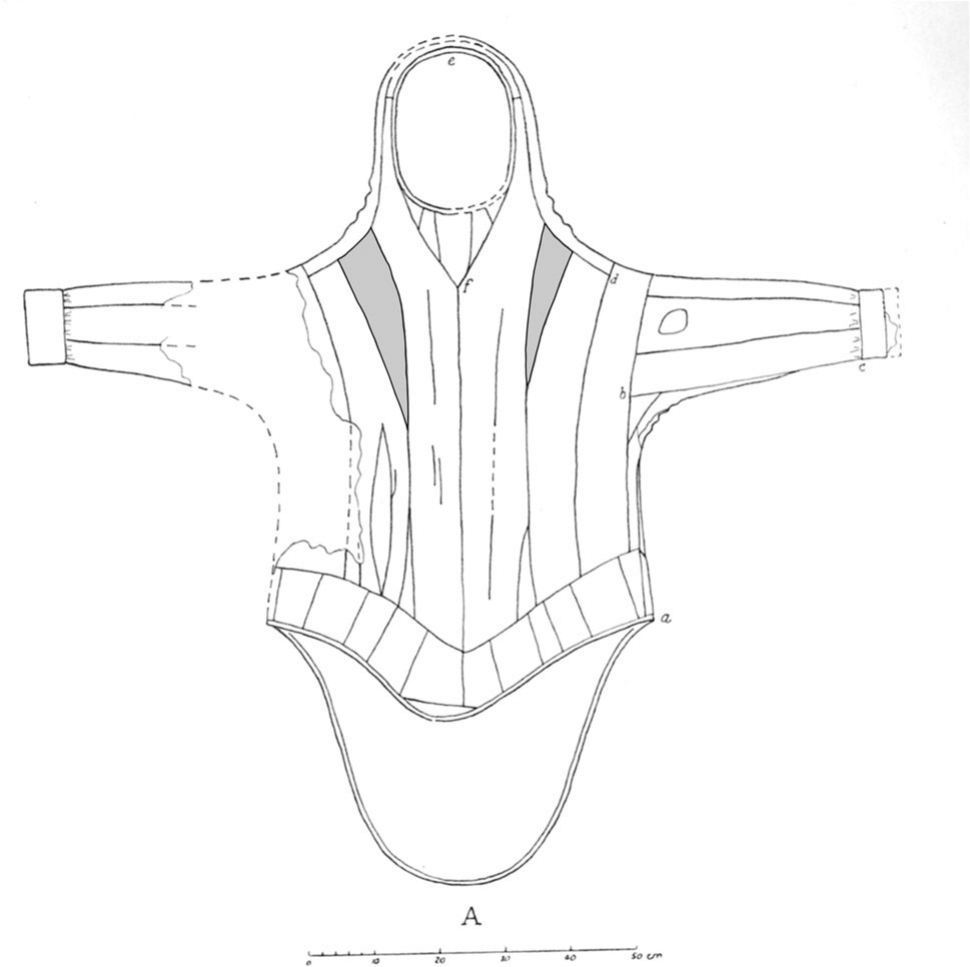
One of three gut-skin parkas excavated in House 28 at the Nuulliit site modified after Holtved (1954, Fig. 49)

One of the key sites to our understanding of the early Inuit history in the North Water area is the abovementioned Nuulliit site. A number of unique artefacts have been excavated at the site, among which are large pieces of three gut-skin parkas (waterproof coats) from the 14th century, uncovered in House 28 (Fig. 5). They are the oldest known gut-skin clothing from the Arctic. A remarkable detail of the tailoring are the elongated wedges sewn into both the front and the back of the parkas (Holtved 1954, Figs. 49–54), which seems to be the predecessor of the so-called ‘double-rooted hoods’ known from 19th and 20th century parkas in Alaska and East Greenland (Hatt 1914). It has been suggested that the double-rooted hood should be seen as representing the head of the walrus with its long tusks (Schmidt et al. 2013). This particular design forms a possible point of entry into the cosmology of the Inuit world as we meet it also in stories related orally to ethnographers, missionaries and others. As Rasmussen has it: “In former times animals in human form were common… In olden times, too, everybody could easily turn into animals, and until quite recently shamans have had the same powers” (Rasmussen 1932, p. 35).

According to some of these stories the difference between humans and other living beings are shaped by the skin they wear, and in which they can take on the skin of others, as well as borrow properties from each other. When taking on the gut-skin parka, the kayaker not only had the benefit of protecting his body against the water, but also of putting on the ‘walrus slough’, i.e. borrowing the strength of the walrus and its ability to keep its breath for a long time under the water. Further, the walrus figures prominently in the myths and stories written down in Greenland during the 18th–early 20th century. A comprehensive archive with the 2280 online records compiled in “Sagn & Myter” and not least an extensive introduction to this database is available (Sonne 2004a, b). Across the 116 records that make reference to walruses it is clear that they were present in diverse ways; as concrete beings of this world, as spiritual collaborators or opponents, and as emblems of particular emotions and social situations. In some of these stories (Sonne 2004a: IDs 93, 625, 795, 937, 1396, 1426) the walrus—in particular the red old, male, single and dangerous specimen (Hansen 1995)—is positioned as unpredictable and murderous; sometimes walruses are seen as brothers-in-laws or foster sons. In other stories (Sonne 2004a: IDs 126, 152, 160, 279, 340, 382, 1850) walruses are instruments of spiritual travels by lending their skin to the human actors in the stories, who may also be *angakut* or their apprentices. In these stories the walrus slough is referred to as a gut-skin parka, and a *pooq*, which again may mean ‘mother’, ‘uterus’, and ‘veil of birth’ in the secret language of the *angakut* (Sonne 2004b).

In the oral traditions of Inuit from across Greenland and across at least three centuries the walrus was of great spiritual and emblematic significance. Further, its importance is materially manifested in, among others, the 19th century East Greenlandic design of the gut-skin parkas. It thus seems reasonable to assume, by recognising the similar design manifested on the 14th century gut-skin Nuulliit parkas, that aspects of this understanding have historical roots dating back to the earliest Inuit societies in Greenland and in Alaska.

In this context, the archaeological material of the early Inuit Ruin Island-phase, a winter house—House 15 on the Skrælling Island (no. 10)—attracts particular interest. As in many similar dwellings, whalebones were used in building the floor, as well as roof support. Interestingly, House 15 had seven walrus skulls incorporated into the house back walls (Howse 2013), a construction detail occasionally seen in Late Dorset winter houses, but usually not in Inuit context. Extensive use of whalebones in the unique architectural design of early Inuit winter houses have been interpreted as a strong reference to a whale, i.e. one ‘crawls inside the whale’ when crawling into the house (e.g. Dawson and Levy 2006). While we cannot infer a similar interpretation of the walrus skulls in House 15 they certainly imply some cosmological connection between walrus and people during the 14th century ad.

**Fig. 6:**
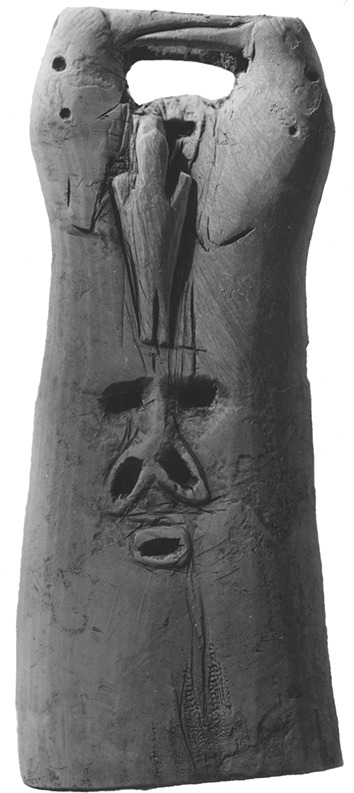
A so-called ‘amulet box’ from the pre-Inuit late Dorset period made of walrus ivory, approximately 95 mm long. Notice the interlocking walrus heads at the top of the carving and the walrus/human being at the side of the box. The amulet box is housed at the Canadian History Museum. Photo: Jørgen Meldgaard

Moving back in time to the pre-Inuit Late Dorset culture a bell-shaped object—usually referred to as an amulet box—made from walrus tusk is of particular interest (Holtved 1944a, plate 1, 26). The box-side has five oval openings indicating eyes, nostrils and mouth, placed in a pattern indicating that the being shown is a mixture of human and animal (LeMoine et al. 1995). Similar box-sides or intact amulet boxes have been found on six other High Arctic Late Dorset sites in Arctic Canada (Appelt 2004). The top section of five of the intact amulet boxes are carved into two opposing walrus figures with interlocking tusks, in light of which it becomes obvious that the merged beings on the sides of the boxes include walrus (see Fig. 6). While carvings of human-like beings, polar bears, and bear-human figures constitute the most commonly portrayed groups of beings among the Late Dorset carvings that have so far been found in Canada and Greenland (e.g. Taçon 1985; Hardenberg 2014), the merged human-walrus beings portrayed on the amulet boxes, suggest that the walrus was an important figure in the Late Dorset cosmology.

The special position of the walrus is further underlined by a unique find from the Alarnerk site, Foxe Basin, Cental Canadian Arctic. The site is among the largest Late Dorset sites, with more than 200 dwellings including middens, of which the majority date to the late pre-Inuit period (c. 1000 bc–1200/1300 ad) (Appelt 2011). It also contains a large number of other features, among which are a number of low mounds originally identified as human burial sites (Meldgaard 1962; Lynnerup et al. 2003). One of these revealed traces of what could have been a complex series of rituals that included the incorporation of child’s jaw-bone and a limb-bone of an adult individual. The focal interment, however, seems to have been the remains of a walrus buried with a number of grave goods, the two human bones, and what is likely to be the remains of a meal cooked upon a small fire-place, placed on top of the walrus (Odgaard 2011).

The abovementioned archaeological finds suggest that there might have been considerable differences between the Inuit and the Late Dorset communities’ understanding of the specific cosmological position of walrus and in what they considered appropriate ways of maintaining these relations. Thus, the relations between humans and walruses may not only have been of even greater economic and social driver among Late Dorset groups in the North Water area and the central Canadian Arctic (Murray 1996, 1999), but also suggest that these relations were inscribed in a complex set of shamanic and shamanistic practices (LeMoine et al. 1995; Appelt et al. 2017). Whether we can establish an entire cosmology or a worldview from the known finds and observations or not, we are certainly allowed to suggest that the walrus was not only an economic resource, but also a significant source of human imagination and reflection.

## Walrus hunting in historical times

Having discussed the prominent position of walrus in prehistoric economy and imagination, we now turn towards more recent times. The first Europeans making it to the Thule region in the 19th century after the receding of the Little Ice Age met a people with limited technologies but with an unquenched thirst for walrus meat (see e.g. Hayes 1867; Peary 1898). The newcomers marvelled at the hunters’ skills at harpooning the huge animals from the ice-edge, and the collective strength needed for dragging them onto the fast-ice or ashore (e.g. Holtved 1967).

The sea ice is still a key factor in the distribution of walrus and in the general population dynamics of the species; this is an increasing challenge, given that the High Arctic ecosystem is also subject to the major repercussions of climate change negatively affecting the walrus hunt (Born et al. 2017, p. 17). From interviews with hunters in the Thule Region, it is clear that the hunters saw “changes in walrus distribution and in the timing of their appearance in the area” (Born et al. 2017, p. 185). More specifically:

A 50-year-old informant from Qaanaaq said that because of climate warming, the sea-ice situation, and the winds, the walruses are now further away from land in October–November and are instead more out in the open sea. A hunter (aged 63) from Siorapaluk stated that the migration routes have also changed. In the 1990s the walruses moved along the coast, but nowadays they migrate farther from the coast. (Born et al. 2017, p. 185).

Other changes were mentioned in Born et al. (2017), not always consistent with each other, but still pointing to a general concern about the walrus and access to it. It is clear that whatever changes are noted, they seem to have hit the inhabitants forcefully over less than a generation, and a degree of nostalgia for past hunting feats is palpable among some of the older hunters. The hunt itself is changing shape and become increasingly individualised with the new technologies. While before, communal hunts would sometimes engage large numbers of hunters in the capture of even a single animal on thin ice (Peary 1898; Holtved 1967), now the hunters set out in their skiffs with just one mate on board (Andersen et al. 2018).

**Fig. 7:**
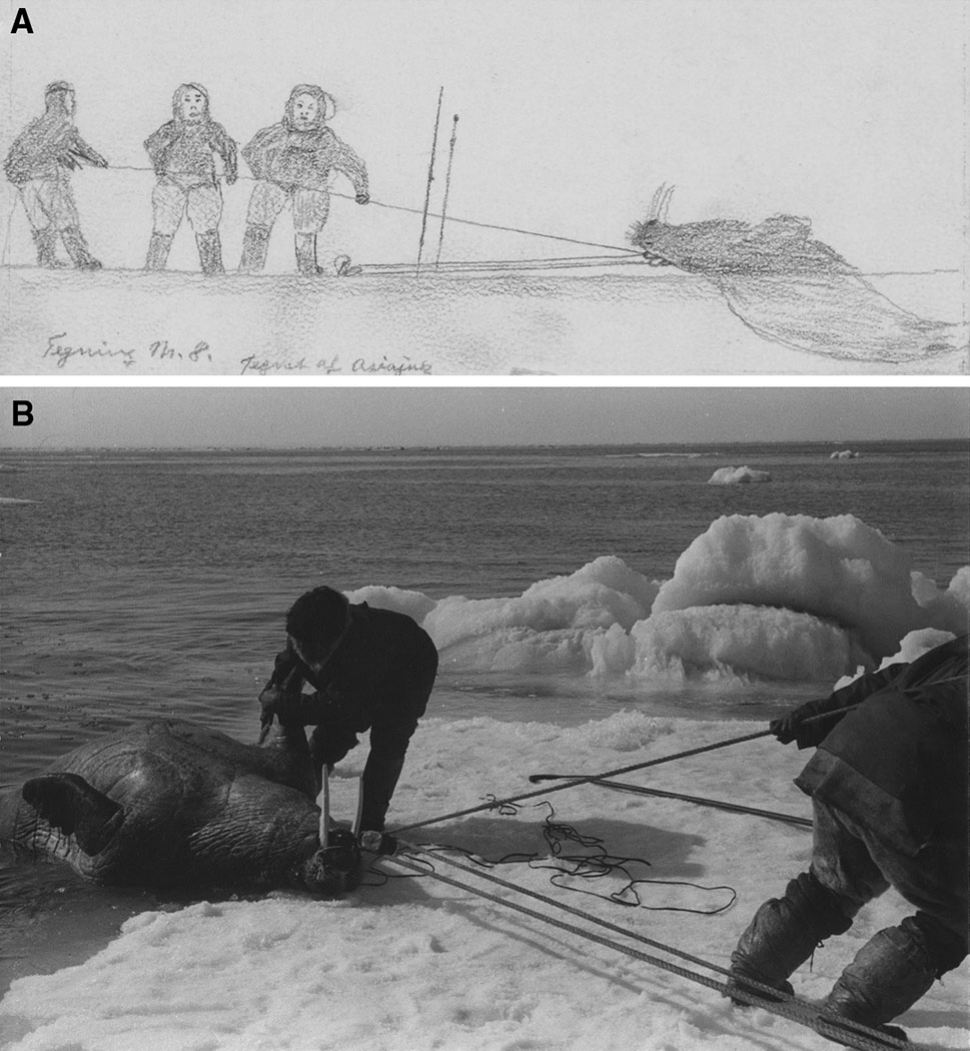
**a**, **b** Walrus caught and pulled onto the Ice. Approximately 50 years and a 1000 km apart. The drawing was done by Asiajuk made in the region of Uummannaq (near the Thule Station) sometime between 1903 and 1920 (Strandgaard 2004). Notice the kayak parkas worn by the three hunters. Courtesy of Ilulissat Museum. The photograph was taken in 1954 in the vicinity of the Igloolik Island (Foxe Basin). The photo is housed by the National Museum of Denmark. Photo: Guy Mary-Rousseliere

**Fig. 8:**
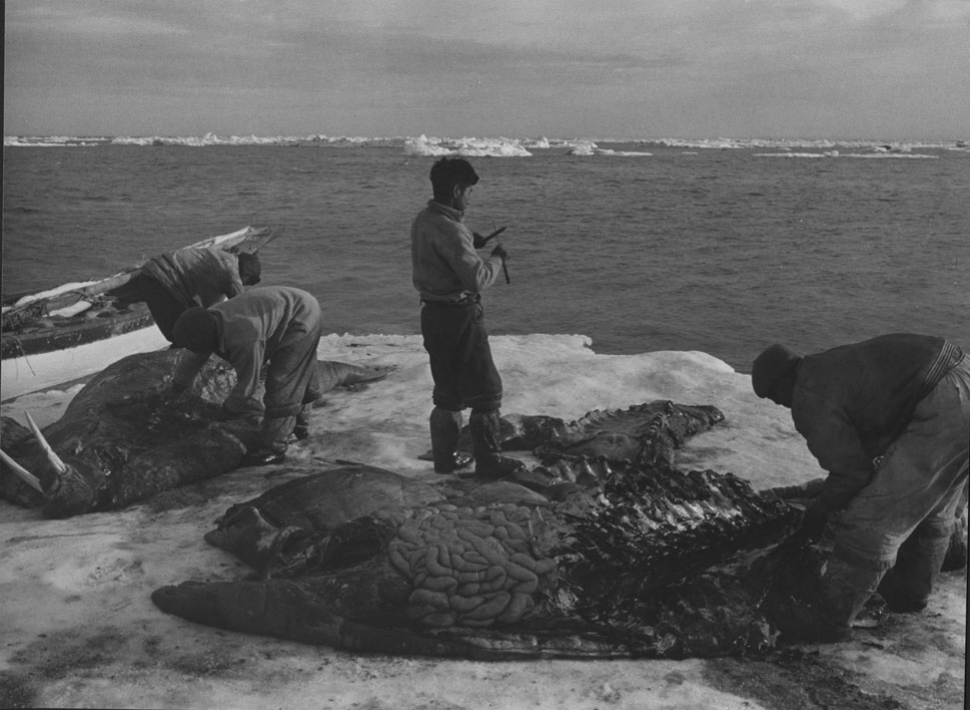
Hunting walrus from ice floes. The photograph was taken in 1954 in the vicinity of the Igloolik Island (Foxe Basin). The photo is housed by the National Museum of Denmark. Photo: Guy Mary-Rousseliere

The walrus hunt on thin-ice was the signature hunt from the 19th century until a generation ago. As vividly described by Holtved (1967, pp. 100–103), the men would go together, bringing their sledges as far out as was considered safe, and then quietly proceeding on the new ice often in a single file, until they spotted a breathing hole, in which they might hear the walrus, or notice some walruses in an opening in the ice. All hunters present would try to put their harpoons into the animal, each man immediately running back a short way on the ice with his line, and fasting it by means of the ice chisel of the lance, which is thrust down into the ice, the end loop of the line around it. The harpooned walrus would immediately go down into the water, and the hunter whose line proved to be the tautest is considered the real harpooner and receives his hunting share accordingly, i.e. first of all head, heart, and entrails (Holtved 1967, p. 102). In reading Holtved’s description, one has to stretch the imagination and seek to picture the scene. For the hunters, however, all of this was part of one comprehensive action, one walrus hunt with no time to stand and ponder the next move. Evidently, the final killing and dragging up of the walrus also was a collective affair, often complicated if the ice was so thin that it was likely to break. Sometimes people would drag it under the water to a place where the ice was thicker, before attempting to drag it up see (Figs. 7a, b, 8).

Clearly, the exact timing and duration of the preferred hunting modes always depended on the ice conditions and the available hunting and transport technologies, and nowadays the spring hunt peaks already during May while the autumn hunt prevails during October–November with only some catch in September (Born et al. 2017). This is due to an earlier break-up of the ice in the spring and later ice formation in winter than just a generation ago, resulting in the walruses leaving the hunting grounds earlier and returning later from their summering grounds. The emblematic walrus hunt on thin-ice has been next to impossible due to the changing ice conditions in recent years (Born et al. 2017).

It is still vividly remembered however; thus in a conversation between an anthropologist (co-author of the present study) and a hunter just a few years ago, the latter dreamily related how ‘before’ they would all go to Pitoqavik (at Cape Chalon) in early winter and work like mad to get as many walruses as possible—although never on the scale described by Peary (1898, I: 421–422)—after which they would fall over in tents on the beach or the ice-foot, and he would fall asleep while others would keep recounting the high points of this particular hunt, and of many others. This storytelling seemed to have been an important part of the framing of the hunt, and European explorers were happy to contribute their stories, once they had taken part.

In Knud Rasmussen’s report from the First Thule Expedition (1912–1914), there is an evocative description of a place, called Neqe, meaning ‘meat’, a headland close to Pitoqavik mentioned above. This is a very telling place-name. For Rasmussen the name testified to the hunters’ pragmatic sense of the good life, right here, at this spot. His description focuses on the summer hunt in the open water: “Out there, in the deceitful ocean, blood-heavy walruses swim about as the living daily bread. They populate the waters, when the brief summer loosens the binds of the ice and opens for the exciting *kayak*-hunt; and true to the millions of mussels at the bottom of the sea they remain over winter, so that the meat caches on land never stand empty on land” (Rasmussen 1915, pp. 14–15). He went on to describe the other advantages of this particular place, so rich in resources and always offering up multitudes of delicacies. The meat was of course the primary blessing, as transpires from the name. Walrus meat was essential not least for the dogs that could travel widely on proper feed—of which walrus meat was prime.

The walrus hunting around the Thule Station (Uummannaq) in the 1930s and 1940s mainly took place on thin-ice with only a light snow cover, often no more than 7–8 cm thick, allowing the walrus to break through it at any point in time as described by Holtved and referred to above. This meant that people had to go rather a long way out towards the open water to find suitable hunting grounds at least in the vicinity of the Thule Station. Yet in other areas, notably around Neqe and Pitoqavik in the north, where Peary had his feast, the ice often broke up close to the coast during the winter storms on the edge of the North Water. After this, the sea would quickly freeze over again in the reigning cold, and Holtved noted how the people from Uummannaq and even from further south, would go up there “during March–April in order to procure meat, when their supplies have been exhausted, and live in snow houses during the time” (Holtved 1967, p. 101).

In 1930, the hunters’ council introduced limitations on the hunt not only around Uummannaq but in the entire region. The council, composed of representatives from the entire region, as well as the Thule Station manager, the priest, and the doctor at Thule ruled that time had come to limit the exploitation of walrus and eiders in particular so as not to deplete the stock. “In our land, it is particularly important to protect eider ducks, arctic foxes and walruses against extinction, and any free hunter should be pleased to go along with such protective measure, because these animals otherwise would be extinct, when those people, who are children now, become adult” (*Kap York Stationen Thules Love af 7. juni* 1929, p. 96).

Part of the problem was the advent of rifles and the massive slaughter of walrus that occurred in the wake of 19th and early 20th century exploration in the region, severely threatening the walrus stock in Northwest Greenland, and even today—and in spite of recent protective measures—the stock is still below its historical abundance. It is recovering, however, partly in consequence of a more strict regulation, such as the quota-system introduced in 2006, leading to a c. 30% reduction in the catch (Born et al. 2017, p. 17). Other factors may also have affected the size of walrus catches, such as a decreasing demand for walrus products, demographic changes, and changes in sea-ice cover (Born et al. 2017, pp. 7–8). Clearly, the hunters who still need a lot of meat, not least for their dogs, are affected by these factors. In the recent interview material collected by Born et al. (2017) one gets a vivid picture of the unrelenting dream of walrus on the one hand, and of the natural and political realities that puncture it on the other. The walrus hunt also still has its dangers; only a couple of years ago two hunters drowned during individual walrus hunts from their kayaks, having been tipped round by the wounded walruses; this caused a lot of local talk and also hit the national news, as witnessed by the anthropologist present. In fact, drowning was always the single most fatal occupational hazard for the Thule hunters (Gilberg 1976, pp. 27–28).

In addition to the shrinking sea ice, another effect of the general warming in the region are the ever more precarious storage facilities; until recently (and even today) people would store the surplus meat in stone caches on cool slopes, where the stone cover would protect it both from stray animals (such as foxes) and from thawing (Born et al. 2017). Now, the natural deep-freezer is increasingly unreliable, and storage becomes a serious problem. Neither this, nor the immanent dangers of the walrus-hunt at sea never made the hunters shun it. The liabilities were overruled by the need for meat, and possibly also by the need to make community ‘happen’. The walrus connected people to the animals, and to each other.

## The walrus as a key species in human history in the North Water area

Walrus hunting was a crucial and defining activity in social life along the coasts of the North Water region during the last 1500–2000 years. The sociality among humans extended to the animals that were incorporated into human society, both literally in being eaten, and figuratively by organising communal activities and collective imageries. From the 8th century and well into the 20th century, and across different indigenous communities, archaeological and historical records alike attest to a communal organisation of the walrus hunt, and a communal distribution of the catch. Walrus was not only an important source of food for humans—and in later periods for their dog teams—it was also a crucial source of raw materials. The intensity of walrus hunting varied greatly within the region, as testified by the diversity of the studied sites, depending on the proximity of the site to good walrus hunting grounds and on available hunting techogies. At some sites from the early Thule period, the Ruin Island-phase, walrus ranked second to only bowhead whale in terms of providing the inhabitants with meat, blubber, and other resources. Importantly, the walrus was far more predictable than the bowhead whale, and would have been the cornerstone in the economy.

Historical sources from the 18th to the 20th century suggest that the walrus was seen as a powerful helping spirit and/or opponent, while the walrus skin was inscribed in a cosmology where the birth veils of humans, the hunting bladder, the gut-skin anorak, and the spiritual journeys of the *angakok* were mutually connected through shared attributes. While we cannot follow these modes of thinking about the human–animal alignment beyond the historical sources, the prehistoric traces rendered available by archaeologists strongly suggest that the walrus played a significant, if variable, role in the imagination of people across the last 2000 years at least. The idea of mutuality and human-walrus interrelatedness is still expressed among Arctic peoples e.g. the Qiqiqtamiut in southeastern Hudson Bay through the idea of all species, animals and humans, having a limited supply of souls that circulate in a closed circle of birth, death and rebirth (Guemple 1994). In such worldview, animals are finite and renewable at the same time, and among present-day Inuit and Inughuit hunters the living resources are understood as not being immediately depletable (see further discussion in Andersen et al. 2018).

While the population size, demography, and behaviour of the walrus of the North Water were markedly influenced by Europeans and eventually by Inughuit after the introduction of guns from the late 19th century onwards, the archaeological record from the North Water area gives no indication of similar effects during prehistory. Only a handful of sites exhibited high exploitation rates and, generally, prehistoric walrus hunt did probably not pose any threat to the walrus populations, although different age classes and both sexes, possibly with higher ratios of juveniles and females than presently, were targeted. Inter-annual and inter-decadal variability in climate resulting in variable ice coverage and storminess, and therefore variable access to the walruses most likely prevented over-exploitation of the walrus stocks. Prior to the introduction of guns fewer animals were probably struck and lost because they were harpooned and secured. Still, it has been suggested that in areas with massive settlements combined with intensive exploitation some long-term depletion of stock may have occurred locally, e.g. in the Foxe Basin by Early and Middle Dorset groups (Murray 1996). In order, however, to prove such possible local effect we need archaeological faunal assemblages with a higher resolution concerning walrus demographic and skeletal profiling combined with paleo-genetic studies.

The most significant aim of this article has been to show the deep continuities between the past and present position of the walrus in the Inughuit economy and worldview. Weapons and words may have changed, but the walrus is still there, in the thick of social life in the North Water region, and as an emblem of continuity across time and space. It is in this sense that we have ventured to speak of the walrus as a ‘key species’—a great practical and imaginative connector between the human and the animal world.

## Footnotes

1 Marine materials are notoriously difficult to radio-carbon date, and in particular whale bone poses a challenge. Using the standard marine correction reservoir age of 400 years the death of the whale seems to have taken place a century or two prior to the use of the *qassi*. A broader marine correction reservoir age (see e.g., Dumond and Griffin 2002, Table 2) would have implied contemporaneity between the bowhead whale cranium and the Nuulliit *qassi*.
